# Growth Hormone and Brain Regeneration: Evidence from Clinical Studies in Dementia, Traumatic Brain Injury, and Stroke: A Systematic Review

**DOI:** 10.3390/ijms27104521

**Published:** 2026-05-18

**Authors:** Vittorio Emanuele Bianchi, Lily Castellar Visbal, Jesús Devesa

**Affiliations:** 1 Clinical Center of Nutrition and Endocrinology, University of San Marino, 47895 Falciano, San Marino; 2 Department of Neurology, Universidad de Cartagena, Cartagena 130001, Colombia; 3 Department of Physiology, Scientific Direction, Medical Center Foltra, 15886 Teo, Spain

**Keywords:** growth hormone, brain regeneration, Alzheimer’s disease, dementia, neuron aging, traumatic brain injury, stroke, neural stem cell

## Abstract

Growth hormone (GH) and insulin-like growth factor 1 (IGF-1) play essential roles in the brain, influencing neuronal and dendritic growth, as well as neurotransmission. These effects persist throughout life. Numerous studies in animals and humans have demonstrated the beneficial effects of GH therapy on memory and cognitive function, as well as on the restoration of neuronal function following injury. All nerve cells, including neurons, glia, endothelial, epithelial, and perivascular cells, are affected by the actions of GH/IGF-1. IGF-1, in particular, has been associated with cognitive function. The GH-IGF-1 axis increases the proliferation of neuronal progenitor cells and the formation of new neurons, oligodendrocytes, and astrocytes. In this study, we searched databases such as PubMed, Google Scholar, and Embase for human clinical trials evaluating the effect of growth hormone (GH) therapy on dementia, Alzheimer’s disease (AD), post-traumatic brain injury (PTI), and stroke. The following search terms were used: “GH and dementia,” “GH and Alzheimer’s disease,” “GH and TBI,” and “GH and stroke.” Inclusion criteria were all randomized controlled trials and observational studies. Exclusion criteria included the lack of cognitive and memory assessments. We found 28 articles. Most studies show the beneficial effects of GH therapy on memory and recovery of brain function after traumatic injury and stroke; however, consistent data are still lacking. The limited number of clinical trials, the small number of patients, and the lack of data on plasma levels of sex hormones that clearly contribute to brain function are limiting factors. This is the case, for example, with androgens. Other critical factors are dosage and treatment duration. Prolonged administration and supraphysiological doses are more effective in inducing positive clinical changes. Growth hormone (GH) therapy is a very promising intervention for preventing and treating dementia and early-stage Alzheimer’s disease, and it contributes significantly to the recovery of brain function in patients after traumatic injury and stroke. Further studies with more robust methodologies are needed to confirm these results.

## 1. Introduction

Growth hormone (GH) is a polypeptide hormone secreted by the anterior pituitary gland. The liver is classically considered its target organ, where it stimulates the production of insulin-like growth factor 1 (IGF-1). The GH-IGF-1 axis has a direct effect on various tissues, primarily regulating longitudinal bone growth and bone metabolism [1], but also on the kidney [2] and muscle metabolism [2,3]. GH is not secreted solely by the pituitary gland. It is now known that GH is expressed in various extrapituitary tissues, such as nervous, ocular, reproductive, immune, cardiovascular, muscular, dermal, and skeletal tissues, suggesting an autocrine action at this level [4,5].

**Figure 1:**
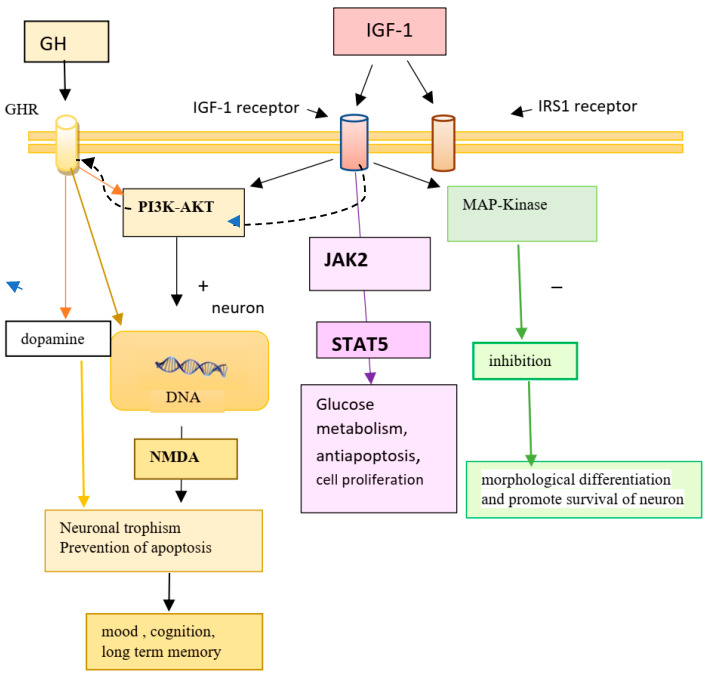
Mechanisms of action of GH and IGF-I at the brain cell level. GH and IGF-1 have an independent genetic activation pathway. GH regulates the transcription of specific genes necessary for these processes. IGF-1 binds to its receptor (IGF-1r) and the insulin receptor (IRS), which induce the action of three pathways: the JAK-STAT, PI3-AKT, and MAPK kinase pathways. GH directly regulates the JAK2-STAT5 signaling pathway. JAK2 receptors have been identified as a tyrosine kinase associated with GHR and other receptors of that superfamily. The JAK pathway is an important transcription factor-dependent pathway regulated by GH. STAT5-mediated genetic regulation is associated with growth by interacting with DNA. The MAPK pathway plays a crucial role in GH signaling. GH treatment leads to neuronal proliferation. MAPK is activated by JAK2-mediated phosphorylation. NMDA receptor activation contributes to long-term memory by enhancing synaptic efficiency in the hippocampus. Dopamine signaling plays a key role in maintaining and restoring neuronal trophism, which is dependent on NMDA receptors.

Many of these local actions are physiologically significant and may be implicated in various diseases, particularly cancer. In this case, for example, autocrine GH is believed to be more oncogenic than pituitary GH [6]. IGF-1, for its part, is also synthesized in the brain, in neurons and glial cells, and exerts regulation over cerebral neuroendocrine regions [7]. GH and IGF-1 are crucial regulators of growth and metabolism. GH interacts with the GH receptor (GHR), a receptor of the cytokine superfamily, to activate the cytoplasmic tyrosine kinase Janus kinase 2 (JAK2) and initiate intracellular signaling cascades. IGF-I, produced in part in response to GH, binds to the heterotetrameric IGF-1 receptor (IGF-IR), an intrinsic growth factor receptor with tyrosine kinase activity that triggers proliferation, anti-apoptosis, and other biological actions. Previous in vitro and overexpression studies have suggested that JAKs can interact with IGF-IR and that IGF-I stimulation can activate JAKs (Figure 1). Huang et al. [8] investigated the interactions between GHR-JAK2 and IGF-IR signaling. Their results suggest that GH-induced GHR-JAK2-IGF-IR complex formation is governed by GH-dependent conformational changes in GHR and/or JAK2, and demonstrate that GH and IGF-I can act synergistically in acute aspects of signaling, but also that IGF-1 enhances the formation of a complex that includes GHR, JAK2, and IGF-IR in these preadipocyte cell lines [8].

GH and IGF-1 share a complex physiological relationship, as demonstrated in β-cell function.

IGF-1R increases GH signaling by regulating the activation of the GHR/JAK2 complex, and soluble IGF-1R is considered to act as a dominant-negative inhibitor of this IGF-1R-mediated increase [9].

GH and IGF-1 regulate development and cell function throughout the body and are considered major regulators of cell function [10]. GH administration (2 mg/day) does not adversely affect body composition or the metabolic profile of small-for-gestational-age (SGA) children receiving medical care at the onset of puberty. A dose-dependent effect on the standard deviation score (SDS) of fat mass (height), trunk fat percentage, and lean mass was observed [11]. GH-induced lipolysis appears to be the most important determinant of GH’s anti-insulin actions, inhibiting insulin-stimulated glucose uptake, particularly in muscle [12].

The GH-IGF-1 axis is a crucial neurotransmitter, neurotrophic agent, and metabolic effector that supports the development of more specific molecular and clinical tools in the brain [13].

GH synthesis in engram cells is necessary for their maturation in the mouse dentate gyrus. Engram cell activity is both necessary and sufficient for memory retrieval. Maturation occurs in a learning-dependent manner, thereby strengthening their functional attributes and contributing to engram formation. Growth hormone is required for the maturation of hippocampal engram cells. The molecular mechanisms driving cell maturation in engrams and hippocampal learning in mice have been explored [14]. Growth hormone deficiency (GHD) causes adverse effects on brain function, which can be reversed by GH replacement therapy [15]. GHD plays a crucial role in reducing tissue and organ homogeneity during normal human development or following injury. IGF-1 transcription depends on adequate GH secretion, but in many tissues, it occurs independently of GH [16]. Multiple subcortical and cortical structures, as well as regional neuronal activity, may be influenced by serum IGF-1 levels [17].

Growth hormone (GH) therapy in children and adolescents with GH deficiency significantly increases body growth, bone development, and brain function [18]. Mood disorders associated with GH deficiency are generally reversed with GH replacement therapy [19].

The GH-IGF-1 axis exerts a major effect on brain development and function as early as the first months of life [20]. GH stimulates cell growth, proliferation, differentiation, neuroprotection, and survival, and influences behavior and cognition [21]. New neurons are generated from progenitor cells in the dentate gyrus of adults, and the human hippocampus can generate neurons throughout life [22]. Aberg et al. [21] first demonstrated that peripheral administration of GH increases the generation of new brain cells in normal adult female rats. IGF-1 is also involved in neurogenesis in mammals [23].

All nerve cells, including neurons, glia, oligodendrocytes, endothelial, epithelial, and perivascular cells, are potential targets of IGF-1, which stimulates synaptic plasticity, a key goal for recovery during cognitive decline [24]. During aging, changes in the functional interactions between the major cell types (neurons, astrocytes, and oligodendrocytes) are known as central nervous system plasticity. All these cells are affected by GH and IGF-I in different ways. In mice, GH has been shown to have a direct effect on neurodevelopment through the activation of neural stem cells [25]. Neural stem cells are also activated by voluntary exercise in a GH-dependent manner [26]. GH receptors and prolactin also play an important role in promoting neurorestorative mechanisms by acting on neural stem cells [25]. Neural stem cells are expressed in multiple brain regions, including the hypothalamic and brainstem nuclei, the amygdala, and the hippocampus [10]. IGF-1 and GH act together in neuroregenerative processes and are collectively referred to as the GH-IGF-1 axis. These effects of GH and IGF-1 are mediated through different pathways (Figure 1).

There is evidence that GH and IGF-1 affect cognitive functions and maintain physiological processes in the adult brain. In fact, declining IGF-I levels may contribute to age-related cognitive decline. Thus, plasma IGF-1 levels in healthy older adults are positively associated with Mini-Mental State Examination (MMSE) scores [27]. Similarly, IGF-1 levels in healthy older men were associated with better performance on tests assessing the effects of aging, particularly information processing speed [28], verbal fluency, and the MMSE [29].

IGF-1 has been shown to increase progenitor cell proliferation and the formation of new oligodendrocytes, blood vessels, and neurons in the dentate gyrus of the hippocampus [30]. In particular, IGF-1 stimulates neuroprotection, development, and differentiation of the central nervous system [14]. In humans, GH levels decline progressively after age 40 and are associated with cognitive decline, underscoring the important role of GH in maintaining cognitive health [31].

Plasma GH levels have been established to decrease by approximately 10–15% per decade after age 30 [31]. Individuals over 60 years of age have frequently been diagnosed with growth hormone deficiency (GHD), with consequent changes in body composition (loss of fat-free mass) and a decline in overall well-being and cognitive function [32]. In humans, a relationship has been found between cognitive impairment and mood disorders in GHD [33]. The aging process primarily affects learning and the formation of new memories [34]. There is a correlation between differences in brain size or the number of neurons and cognitive abilities across species. Cognition is the result of a global neural network [35]. The cognitive process is primarily regulated by the hippocampus [36]. Gene expression studies have shown that the dorsal hippocampus, which corresponds to the posterior hippocampus in primates, primarily supports cognitive functions [20], while the ventral hippocampus is correlated with regions involved in emotion and stress. The hippocampus is a brain region capable of neurogenesis, a process that involves the continuous generation of neurons during adulthood [22].

Another brain region where neurogenesis continues into adulthood is the subventricular zone (SVZ) of the lateral ventricle. With aging, both regions are negatively affected, as evidenced by reduced neural stem cell (NSC) activation [21], a decrease in the number of neurons generated in adulthood, and deficits in cognitive function [37].

This review aims to evaluate the clinical effects of GH on brain function in dementia and AD, and on recovery after TBI and stroke.

### 1.1. Mechanism of Action of GH on the Brain

Growth hormone (GH) administration has a direct effect on the brain because it can cross the blood–brain barrier (BBB) and pass from the circulation into the cerebrospinal fluid (CSF) [38], interacting directly with growth hormone receptors (GHRs). The presence of GHRs in the brain was first discovered in 1993 [23], with higher expression levels during neurogenesis and brain development. The distribution of GHRs in the brain is crucial, supporting the role of GH in central nervous system (CNS) physiology. GHRs have been identified in cortical cells of embryonic rat brains, regulating the proliferation and differentiation of cortical cells and the dentate gyrus of the adult hippocampus [39]. In target cells, GH activates several signaling pathways, including the Janus kinase (JAK)/signal transducer and activator of transcription (STAT) pathway. This process is regulated by suppressors of cytokine signaling proteins (SOCS) [25]. Activation of pSTAT5 produces its metabolic effect in most areas of the telencephalon, hypothalamus, and brainstem [40]. GH induces pSTAT5 immunoreactivity in neurons, but not in astroglial cells [41].

GH administration alters the cerebrospinal fluid (CSF) concentration of some brain neurotransmitters important for mood and cognition, such as homovanillic acid (HVA), a dopamine (DA) metabolite, and aspartate, an excitatory amino acid that acts as a ligand for the N-methyl-D aspartate (NMDA) receptor [42,43,44] (Figure 1).

Local IGF-1 production mediates these responses [39]. Furthermore, ghrelin/GHSR (growth hormone secretagogue receptor) signaling maintains hippocampal integrity and exerts an inherent neuroprotective effect [21,45].

CNS plasticity is characterized by changes in the functional interaction between major cell types: neurons, astrocytes, and oligodendrocytes. IGF-1 increases progenitor cell proliferation and the formation of new neurons, oligodendrocytes, and blood vessels in the dentate gyrus of the hippocampus [21].

GH functions as an autocrine mitogen in adult neurosphere cultures, also promoting neural progenitor cell proliferation and neurosphere culture self-renewal [46,47]. Aberg et al. [48] demonstrated that GH administration stimulates the generation of new brain cells in normal adult female rats, not only under GH-deficient conditions but also under physiological conditions. Devesa et al. [49] demonstrated, in mice, that GH treatment can improve the proliferation and survival of neuronal progenitor cells, thus promoting brain repair after brain injury.

In aged animals, GH/IGF-1 administration increases neurogenesis, vascular density, and glucose utilization, all processes involved in learning and memory [33].

It is important to note that the properties of neural stem cells (NSCs) of neurogenic radial glia derived from the human fetal forebrain respond to the exogenous administration of GH and prolactin (PRL) and may play a role in brain recovery processes related to injury [25]. Gonadotropin-releasing hormone (GnRH) and GH in the CNS have potential as a combination therapy [50].

Alterations in dopamine (DA) turnover in the dopamine-rich hippocampus can influence memory functions and NMDA receptor activation, contributing to long-term potentiation of synaptic efficacy and, ultimately, memory consolidation [8]. Le Grevès et al. [51] demonstrated that GH administration in rats increases hippocampal mRNA expression for the N-methyl-D-aspartate (NMDA) receptor subunit 2B, improving cognitive abilities. This finding indicates that GH can affect the hippocampal NMDA receptor both directly and indirectly (via aspartate).

In adult patients with GH deficiency, decreases in the levels of N-acetylaspartate (NAA), a marker of neuronal density and integrity, and choline, a marker of membrane synthesis, were observed [52]. Therefore, low IGF-1 levels may be associated with neuronal damage.

In elderly subjects, low IGF-1 levels were associated with a poor prognosis after ischemic stroke [53]. This finding suggests that circulating IGF-1 may protect against damage to neurons injured by ischemia. IGF-1 is effective for recovery after stroke, as demonstrated by the results of studies in patients during post-ischemic stroke rehabilitation. Improvements in functional and cognitive scores, as well as favorable outcomes, were associated with higher IGF-1 levels [54,55]. Finally, IGF-1 enhances acetylcholine release from the hippocampus [56].

GH and IGF-1 replacement therapy in aged animals increased neurogenesis, vascular density, and glucose utilization, and altered the composition of NMDA receptor subunits in brain areas involved in learning and memory. Sonntag et al. [57] demonstrated, in aged animals, that IGF-1 therapy increased levels of the N-methyl-D-aspartate receptor, one of the key receptors in the human brain. Radioligand analysis revealed that the highest receptor density is found in the hippocampus, striatum, cortex, and amygdala. Associative memory, learning, and synaptic density are directly related to the efficient functioning of the NMDA receptor [58].

Recent discoveries have provided evidence of the contribution of the GH-GHR-IGF1 axis to mitochondrial biogenesis, mitophagy (or autophagy), and mitochondrial function under various physiological conditions [59].

GH is involved in brain repair following injury, and several studies have shown that its administration after injury confers neuroprotection and accelerates the recovery of a range of neuronal functions [60].

### 1.2. GH/IGF-I Axis and Dementia

There is evidence of a relationship between declining GH and IGF-I levels and age-related changes in body composition and physical function. The potential role of IGF-I levels in the development of dementia has been examined. The decline in plasma IGF-I levels with aging is correlated with cognitive decline [27]. For example, IGF-1 and IGF-binding proteins (IGFBPs) have been studied in patients with Alzheimer’s disease (AD). AD patients had lower levels of IGF-1 and IGFBP-3 and higher levels of IGFBP-1 compared to controls.

From this, it has been proposed that IGF-1 enhances amyloid clearance, which could identify therapeutic targets in Alzheimer’s disease [61,62]. Animal studies have shown that GH administration in GH-deficient rats [54] significantly reduced cognitive decline, improving spatial learning and memory [63].

In patients with growth hormone deficiency (GHD), cognitive deficits [34], difficulty concentrating, reduced psychological well-being [35], and impaired spatial learning [36] have been observed. Similar findings have also been observed in an animal model [64]. Memory impairment and subnormal IQ scores were associated with low IGF-I levels, indicating that subnormal cognitive performance was specifically related to GHD [33]. The onset of GH deficiency in childhood affects brain substrates of selective attention associated with anterior cingulate cortex function [65].

This suggests that GH and/or IGF-1 are essential for brain development during childhood and possibly even prenatally [52]. Indeed, in congenital GH/IGF-1 deficiency, subnormal brain size has been observed, reflected in a smaller than normal head circumference [66]. Furthermore, adolescents and adults with GH insensitivity (Laron syndrome, an inherited disorder that causes a lack of IGF-1 production) appear to suffer from intellectual and cognitive deficits [67] and exhibit brain abnormalities detected by magnetic resonance imaging [68].

Memory is stored in specialized cells known as engram cells; GH is required as a key mediator of the maturation of hippocampal engram cells [14].

A correlation was observed between IQ and brain abnormalities resulting from molecular defects in the GH receptor gene in all Laron syndrome patients with IGF-I deficiency [69].

## 2. Methods

**Figure 2:**
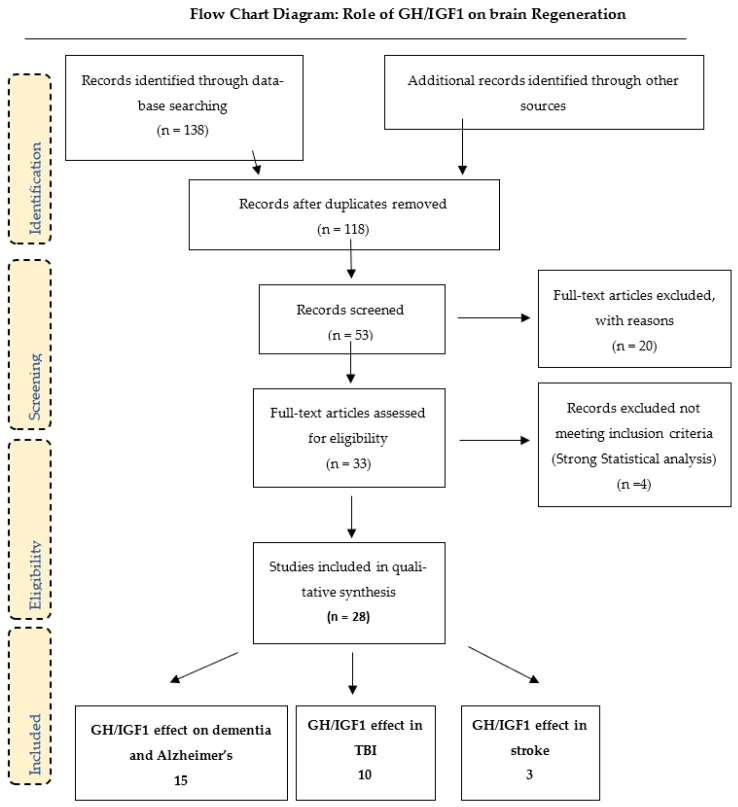
Flow chart showing the methodology of selection of the articles.

A comprehensive search was conducted in PubMed/MEDLINE, Google Scholar, and Embase using a peer-reviewed search strategy. Studies were selected. Eligible studies included clinical trials, observational studies, and case series, with the following keywords: “growth hormone” and “Alzheimer’s disease”; “growth hormone” and “dementia,” with or without growth hormone deficiency (GHD); “GH and TBI”; and “growth hormone” and “stroke.” See the flowchart in Figure 2. Inclusion criteria were randomized, double-blind crossover studies, long-term clinical trials, and studies that included cognitive testing. Exclusion criteria were the lack of memory assessment and cognitive testing.

### 2.1. Protocol and Registration

The present review followed the Preferred Reporting Items for Systematic Review and Meta-Analysis (PRISMA). The protocol of this systematic review was submitted for registration in the International Prospective Register of Systematic Reviews (PROSPERO) under registration number 1347643.

### 2.2. Risk of Bias

In the included studies, the SYRCLE risk of bias assessment indicated that most domains were classified as low risk. This suggests that the articles met and reported the methodological criteria for the handling of human subjects in general.

## 3. Results

In the systematic review, we retrieved fifteen selected studies on GH administration in mild cognitive impairment and Alzheimer’s disease [70,71,72,73,74,75,76,77,78,79,80,81,82,83,84,85], as shown in Table 1. Ten studies that evaluated the effect of GH in patients after a traumatic brain injury [86,87,88,89,90,91,92,93,94,95] are presented in Table 2, and three studies that evaluated the effect of GH in patients after a stroke are presented in Table 3. The logical sequence of the arguments in the articles relates to the number of clinical studies retrieved by publication date.

Most studies showed improvement in cognitive function and memory [70,71,72,73,74,75,76,77,78,79,80], while others did not [81,82,83].

Devesa et al. [80] were the first to demonstrate the positive effects of GH (0.4 mg/day, subcutaneously for three weeks) on brain metabolism in a patient with mild cognitive impairment (MCI) (ApoE 3/4 genotype) without GH deficiency, restoring function in affected areas related to cognition, memory, and behavior. The study was complemented by a PET-SCAN analysis. That same year, Arwert et al. [84] showed subnormal working memory speed, especially under heavy workloads, which was established using functional magnetic resonance imaging (fMRI).

Almqvist et al. [72] reported improved memory function after four weeks of GH treatment in five patients with childhood-onset growth hormone deficiency (CIGH). An uncontrolled study by Sartorio et al. [71] showed an improvement in intellectual functioning in eight patients with combined growth hormone deficiency (CGD) after six months of growth hormone (GH) therapy. These results were partially confirmed in a study of 48 men with combined growth hormone deficiency (CO-GHD), using a 6-month placebo-controlled design followed by a 10-year uncontrolled period of growth hormone (GH) treatment. No effects of GH treatment were observed during the placebo-controlled phase in patients with normalized serum IGF-1 levels.

However, a 12-week, double-blind, placebo-controlled crossover study of GH treatment in six patients with GH deficiency showed no beneficial effect of GH replacement therapy on cognition [85].

Bengtsson et al. [73] reported significant improvements in body composition, strength, and mental function in a 26-week, double-blind, crossover study.

An improvement in memory function was observed after one year of therapy, and plasma IGF-1 levels normalized, a change that was maintained at 10 years of follow-up, suggesting that GH therapy should be continued for an extended period to maintain cognitive improvement and prevent relapse [70]. An improvement in memory function was also observed in CO-GHD patients treated with GH for at least one year [96], with no improvement observed during the first year of GH treatment, providing further support for the minimum one-year treatment period required to improve cognitive function.

However, the beneficial effects of GH treatment in adult patients require further investigation and should be conducted in a large population. Other placebo-controlled studies by Degerblad et al. [85] and Deijen et al. [75] indicate the absence of any effect of GH treatment, except during six months of supraphysiological treatment. Therefore, administering supraphysiological doses of GH may enhance the effect of GH replacement in adults with CO-GHD. Baum et al. [82], in a placebo-controlled study of GH-deficient men, found no improvement in cognitive performance or memory after 18 months of GH replacement at a dose of 4 +/− 2 µg/kg per day. Soares et al. [77] analyzed 9 adults with GHD (2 with late-onset GHD, attention and memory, and 7 with adult-onset GHD (AO-GHD)) before and after 6 months of GH treatment in a placebo-controlled trial. After 6 months of therapy, significant improvements were observed in a variety of tests of cognitive function, including memory. Partly in line with these findings are the results of an 18-month placebo-controlled study that reported improved attention after at least 3 months of GH replacement. However, even after 18 months of GH treatment, the improved attention was not accompanied by an improvement in memory performance [76]. In a parallel placebo-controlled study, Sathiavageeswaran et al. [78] demonstrated that patients receiving GH for 12 months experienced significant cognitive benefits compared with the control group, whereas after 6 months of therapy, the improvement in memory was modest. This study underscores the importance of the duration of treatment. The participants were men or women aged 60 to 80. However, the significant difference between the GH and control groups was partly due to a decline in performance in the placebo group. Most studies have shown that GH therapy has a beneficial effect on brain function, but some controversies remain. Therefore, the beneficial effects of GH therapy on cognition appear to require further investigation. In adult patients with GH deficiency, verbal memory, as measured by the 15-word recall score, and planning behavior, processing speed, and attention, as measured by the trail tracing test, were impaired [52]. In Prader–Willi syndrome, where patients present with intellectual disability, GH treatment after 6 months showed a significant improvement in cognition and reaction time [43]. This study evaluated 9 women and 10 men with a mean age of 25 years. In another study, three years of GH treatment maintained full-scale, verbal, and performance IQ. Emotional and behavioral disturbances remained stable and were similar compared to peers with other intellectual disabilities. The dose was 33% of the pediatric dose [97]. In conclusion, the prevalence of studies has demonstrated a significant beneficial effect of GH treatment on brain function and cognition in patients of different ages with or without GHD.

### 3.1. The GH/IGF-I Axis and Dementia

There is evidence of a relationship between declining GH and IGF-I levels and age-related changes in body composition and physical function. The decline in plasma IGF-I levels with aging is correlated with cognitive decline [27]. For example, IGF-1 and IGF-binding proteins (IGFBPs) have been studied in patients with Alzheimer’s disease (AD). AD patients had lower levels of IGF-1 and IGFBP-3 and higher levels of IGFBP-1 compared to controls [67]. IGF-1 levels are inversely correlated with cognitive decline [68] and directly correlated with cortisol. Devesa et al. [98] demonstrated in a ten-year-old girl who suffered birth asphyxia with significant cognitive deficits that GH administration specifically improves cognitive function during neurorehabilitation. Consistent with this study, recent research has demonstrated a marked reduction in IGF-1 levels in patients with mild to moderate Alzheimer’s disease (3.7 ± 1.2 pg/mL) compared to healthy elderly subjects of similar age (IGF-1, 9.5 ± 2.8 pg/mL after growth hormone therapy) [96]. In another study, cognitive function was assessed in 49 healthy centenarians (mean age, 100.4 years) using clinical dementia assessment scales, and those with lower IGF-1 levels had a higher incidence of dementia. Low serum IGF-1 levels may contribute to the progression of dementia in centenarians [99]. IGF-1 modulates the clearance of β-amyloid from the brain, and β-amyloid deposition increases with decreasing IGF-1 levels.

Therefore, it has been proposed that IGF-1 enhances amyloid clearance, which could identify therapeutic targets in Alzheimer’s disease [61,62]. Animal studies have shown that GH administration in growth hormone-deficient (GHD) rats [54] significantly reduced cognitive decline, improving spatial learning and memory. The cognitive deficit, evidenced by a decrease in spatial learning ability, was reversed [63].

In adult patients with GH deficiency, verbal memory, measured by the 15-word recall test, and planning, processing speed, and attention, measured by the scanning test, were impaired [52]. That same year, Arwert et al. [84] reported subnormal working memory speed, particularly under heavy workloads, as determined by functional magnetic resonance imaging (fMRI) assessments.

This suggests that GH and/or IGF-1 are essential for brain development during childhood and possibly even prenatally [52]. Indeed, in congenital GH/IGF-1 deficiency, subnormal brain size has been observed, reflected in a smaller than normal head circumference [66]. Furthermore, in adolescents and adults with growth hormone insensitivity (Laron syndrome, an inherited disorder resulting in a lack of IGF-1 production), growth hormone receptor (GHR) inhibition appears to have the potential to protect against age-related diseases such as cancer and diabetes. However, it is unknown whether cognitive function and brain structure are affected by age-related cognitive decline [100] and whether they exhibit brain abnormalities detectable by magnetic resonance imaging [68]. A correlation between IQ and brain abnormalities resulting from molecular defects in the GH-R gene was observed in all Laron syndrome patients with IGF-I deficiency [69]. It is reasonable to assume that other, as yet unknown, factors related to molecular defects in the GH-R are the primary cause of the differences in intellectual ability and brain abnormalities.

### 3.2. GH Therapy in Traumatic Brain Injury (TBI)

Moderate to severe head trauma results in hypopituitarism in approximately 40% of patients [101], with a consequent neuroendocrine disorder. Three months after head trauma, hypopituitarism was still evident in 54–56% of patients [102], and it persisted in 36% of patients one year later [91], affecting the gonadotropic axis in 21% of cases, the somatotropic axis in 10%, and the corticotropic axis [103]. The underlying pathophysiological cause of hypopituitarism is believed to be venous infarction of the long hypophyseal portal veins [96]. Aimaretti [104] found an incidence of hypopituitarism after acute head trauma in 33% of patients. Panhypopituitarism, with both multiple and isolated pituitary hormone deficiencies, was observed in 5.7%, 5.7%, and 21.4% of cases, respectively. Pituitary function recovered in 57% of post-traumatic patients one year later [105]. Growth hormone deficiency (GHD) was confirmed in 5% of patients, and 19% showed below-average GH levels [106]. Patients with GHD after a traumatic brain injury (TBI) showed greater deficits in attention, executive function, and memory than those without GHD [87]. Devesa et al. [107] demonstrated that GH can be combined with rehabilitation to improve disabilities in TBI patients. Therefore, these cognitive impairments in patients who develop GHD after a TBI may be reversed with GH treatment. We selected nine clinical trials that evaluated the effect of GH administration in patients after TBI, recruiting 440 patients with a mean age of 44.2 ± 9.5 years. All studies demonstrated significant improvements in quality of life, memory, cognition, and motor control [86,87,89,90,91,92,93,94,95,107,108,109]. The studies are summarized in Table 2. Most studies showed improvement in cognitive impairment after GH treatment in traumatic brain injury (TBI) [87,89,90,91,93,94,95,107,110], while only one study found no positive effect of GH therapy [86]. Traumatic brain injury (TBI) has been observed to cause hypothalamic-pituitary disruption and, subsequently, abnormalities in hormone secretion [68,111]. Peak GH appears to be an independent predictor of improved cognitive abilities; a favorable outcome following a TBI is likely associated with a greater GH reserve. High et al. [91] assessed GH deficiency in 83 subjects with moderate to severe TBI, at least one year post-injury, but primarily several years later. Of the 43 out of 83 subjects diagnosed with GHD or GH insufficiency, 23 agreed to be randomly assigned to and treated with either GH or placebo for one year. Twelve subjects received GH and 11 received placebo. The mean age of the placebo group was 39.1 years (SD 8.5), and that of the active rhGH group was 36.1 years (SD 10). Neuropsychological tests were administered before and after treatment. Four measures showed differential improvements between the treatment and placebo groups. The study provides preliminary evidence suggesting that GH therapy improved the cognitive impairments observed in GHD/GHI persons after the chronic phase post-TBI. Furthermore, moderate improvements in processing speed and memory capacity, as well as a marked improvement in quality of life [83], were also observed.

**Table 2:** Effect of GH therapy in patients who suffered traumatic brain injury. 1215 patients. GHD = growth hormone deficiency; ALS functional rating scale revised (ALSFRS-R). WAIS = Wechsler Adults Intelligence Scale IQ = intelligence quotient.

Authors	Patients	Age (Years)	Study Type	Duration	GH Dose (mg/Day)	Clinical Outcome
Dubiel, R. 2018 [86]	63 TBI	16–65	Randomized double-blind study	6–12 months		No differences from the rhGH group and placebo in the Disability Rating Scale, Glasgow Outcome Scale-Extended, or neuropsychological function were found.
Gardner, 2015 [87]	161	42.6	Clinical study	1 year	0.37 mg/day	GH therapy achieved clinically relevant, long-term benefits in quality of life.
Devesa, 2015 [88]	13 TBI patients	6–53	cognitive disorders; motor plegia; neurogenic dysphagia (n = 5), vegetative coma (n = 2) and amaurosis	8 months	1 mg/day, 5 days/week	All patients showed significant improvements during and after the combined treatment.
Moreau, 2013 [89]	23	37.9	Clinical trial	1 year	0.30 mg/day	Improvement in cognition and quality of life
Reimunde, 2011 [90]	11 M (mean 44.5 months after injury)	53.3	Clinical trial	3 months	0.5 mg/day for 20 days, then 1 mg/day for 5 days/week	Significant improvement of cognitive parameters, total IQ, and WAIS scale
High, 2010 [91]	23	39.1	Randomized Controlled Trial	1 year	0.2 mg/d, increasing 0.2/month up to 0.6 mg/d	Significant improvements in cognitive impairments that are partially reversible
Maric, 2010 [92]	6 5 M 1 W	38.6		6 months	0.3 mg for males and 0.4 mg for females sc	GH therapy induced reduction in depression, Social dysfunction, and specific cognitive domains.
Kreitschmann-Andermahr, 2008 [93]	84 GHD (28 childhood)	36.7	Clinical trial	1 year	0.31 mg/d > 0.40	Improvement of the quality of life and final height.
Hatton, 2006 [94]	97		Randomized double-blind study	14 days	IGF-1/GH therapy IGF-1 continuous intravenous infusion (0.01 mg/kg/hr), and GH (0.05 mg/kg/day)	IGF-I and GH produced sustained improvement in metabolic and nutritional endpoints
Rockich, 1999 [95]	23 TBI	18–59	Randomized double-blind study	14 days	HGH 0.05 mg/kg/day or saline for 14 days.rhIGF-1/rhGH reached a peak IGF-1 concentration (1199.3+/−84.0 microg/L	Significant improvement in physical and mental recovery

Patients receiving supraphysiological GH doses showed a more rapid improvement in cognitive function than those receiving lower doses [75]. However, both groups showed a significant improvement in cognition after 12 months of treatment, which was maintained for one year after suspension of therapy.

We can conclude that there is evidence of beneficial cognitive and physical effects, although the controlled nature of the supportive studies requires updating.

Furthermore, Szarka et al. [110] in a well-conducted systematic review, reported on twelve studies with 264 patients who suffered from mild to moderate or severe TBI, that the GH treatment, with gradually increasing doses, induced a moderate improvement in processing speed and memory capacity, decreased the severity of depression, and induced a marked improvement in quality of life.

In a recent systematic review [112], researchers reported that GH administration improved IGF-1 levels, fatigue, mood, physical performance, and cognition, and that it could benefit a wide range of patients with symptomatic TBI. Furthermore, structural and functional neuroimaging changes following GH administration included increased cortical thickness and gray matter volume, as well as improved functional connectivity. Limitations of these studies include the small number of patients and the use of different neuropsychological tests in patient assessment.

### 3.3. GH Therapy After Stroke

The brain is an organ with a high intrinsic capacity to respond to physical and chemical injuries by altering the properties of its neural circuits. This brain plasticity can lead to a wide range of spontaneous recovery. The term “stroke” denotes a brain injury resulting from focal or multifocal ischemia and hypoxia of specific brain areas. Rehabilitation can improve and modulate neuronal plasticity [84]. Stroke is one of the leading causes of mortality and disability in the elderly population worldwide [113]. In 2017, there were 112 million strokes in the European Union, 0.46 million deaths, and 7.06 million people with disabilities. However, despite the high morbidity and mortality rates, a satisfactory therapy has not yet been found. Treatments with mesenchymal stem cells (MSCs), mononuclear stem cells (MNCs), umbilical cord stem cells, and neural stem cells (NSCs) have been investigated, but so far without clear and satisfactory results. Innovative combinatorial approaches involving growth factors and biomaterials, such as injectable hydrogels, are being implemented [85]. Brain regeneration after injury remains active even weeks after a stroke [114,115].

In young adult mice, following the induction of an experimental stroke, biochemical and histological analyses revealed loss of neural tissue, neurotrophic factors, and impaired neuroplasticity. Growth hormone (GH) treatment in stroke mice significantly improved learning performance compared to untreated mice [116]. Furthermore, GH treatment protects against neuronal loss in mice after a stroke. GH treatment resulted in a significant improvement in the levels of neurotrophic factors such as IGF-1 and VEGF (vascular endothelial growth factor) in circulatory and peri-infarct regions. Growth hormone (GH) treatment in stroke mice not only increased protein levels and density of presynaptic markers (SYN-1 [synapsin-1]) and myelination markers (MBP [myelin basic protein]), but also increased the density of vascular markers (CD31 and collagen IV) in the peri-infarct region [117]. GH treatment increased the number of proliferating neuronal precursor cells and post-mitotic migrating neuroblasts after stroke [25]. These findings demonstrate that GH treatment has potentially valid therapeutic effects in patients after stroke.

This is consistent with the finding that neuronal progenitor cell markers, synaptic plasticity, and cerebrovascular remodeling in the hippocampal formation are significantly increased after GH treatment.

Estrogens contribute to IGF-1-mediated neuroprotection in ischemic stroke by interacting with microglial functions [118]. Performance on visual discrimination tasks after stroke improves following GH treatment, providing further neurobiological evidence that this hormone promotes hippocampal plasticity and thus enhances cognitive recovery after stroke [116].

One study demonstrated that long-term treatment with MR-409 (a growth hormone-releasing hormone [GHRH]) (5 or 10 μg/mouse/day) via subcutaneous (s.c.) injection significantly reduces mortality, ischemic injury, and hippocampal atrophy, and improves neurological functional recovery in mice subjected to transient middle cerebral artery occlusion (tMCAO). Furthermore, MR-409 can stimulate endogenous neurogenesis and attenuate tMCAO-induced neuroplasticity loss. MR-409 also enhances proliferation and inhibits apoptosis of neural stem cells treated with oxygen and glucose deprivation followed by reperfusion. The neuroprotective effects of MR-409 are closely related to the activation of the AKT/CREB and BDNF/TrkB pathways. In conclusion, the GHRH agonist MR-409 exhibits remarkable neuroprotective effects by enhancing endogenous neurogenesis in mice with cerebral ischemia; however, clinical studies in humans are limited, as shown in Table 3.

In humans, several clinical studies have demonstrated that GH therapy is associated with a higher survival rate and improved cognitive recovery in stroke patients. Song et al. [119] observed that, in 23 stroke patients, performance and muscle strength increased after 6 months of GH treatment.

Hernández-Bernal et al. [120] demonstrated that patients treated with combined epidermal growth factor and growth hormone-releasing hexapeptide therapy in acute ischemic stroke [120] showed a favorable neurological and functional outcome at 90 and 180 days, as evidenced by inferential analysis of the NIHSS, Barthel, and mRS scales, and a higher survival rate. Feng et al. [121] found that, after 6 months of GH treatment, the mean Montreal Cognitive Assessment score improved significantly, and the GH-treated group showed a significant increase in the activation area with increased amplitude of low-frequency fluctuations (ALFF) in the frontal lobe, putamen, temporal lobe, and thalamus. In summary, GH replacement therapy has a significant impact on global and specific cognitive functions in post-stroke cognitive impairment.

However, these data should be interpreted with caution due to the small sample size, multiple comparisons, and the young age of the participants.

**Table 3:** Effect of GH in patients with stroke. EGF = epidermal growth factor; GHRP = growth hormone-releasing hexapeptide; Korean Modified Barthel Index (K-MBI).

Authors	Subjects	Age (Years)	Duration	Studies Type	Therapy	
Hernandez-Bernal, F.2024 [120]	21 M, 12 h after stroke	66 ± 11 (18–80)	6 months	randomized, open-label, controlled trial	75 μg rEGF + 3.5 mg GHRP6 i.v (n = 10) 75 μg rEGF + 5 mg GHRP6 i.v., (n = 10)	A higher survival rate was observed among patients treated with the combined therapy.
Feng, X. 2020 [121]	60M after three months of stroke		6 months	Randomized controlled trial		Significant impact on global and domain cognitive functions in poststroke cognitive impairment.
Song, J. 2012 [119]	22	60–71	6 months		13 mg/week (Declage)	GH group showed more improvement in the K-MBI score, and felt less tired than the control group, without any sign of harmful effects.

## 4. Discussion

As we have discussed, GH plays an integral role in CNS development and recovery from injury, the aging process, dementia, and brain dysfunction [122].

GH has been shown to increase neuronal regeneration [95], myelin synthesis [123], and dendritic branching. Importantly, GH infusion activated a population of neural stem cells (NSCs), leading to neuronal differentiation in mice [26]. These stem cells are also activated by voluntary exercise in a GH-dependent manner [26]. However, published studies exhibit some bias. GH therapy in adults with GH deficiency has different effects compared to adults without GH deficiency. Ultimately, the dosage and duration of administration are the main determinants. In dementia studies, participants in young adults with growth hormone deficiency ranged from 17 to 68 years of age [74], and GH doses varied from 0.5 mg/day to 1 mg/day, with durations ranging from 14 days to 1 year. In traumatic brain injury (TBI) studies, participants ranged from 6 to 84 years of age, and doses administered ranged from 0.013 to 0.16 mg/day. In the stroke group, data were available from only one study (age 60–71 years and GH dose of 13 mg/week). Despite these physiological differences, most studies reported significant improvements in cognitive function and quality of life. Furthermore, several tests exist to assess changes in multidimensional cognitive functions and psychometric parameters, such as the Mini-Mental State Examination (MMSE), the Short Mental Status Test (STMS), and the Geriatric Depression Scale [112], but different methodologies are used in studies to evaluate cognition. Another critical aspect is that plasma levels of sex steroids were not assessed, despite their high relevance to brain function. In fact, testosterone exerts a significant neurotrophic effect in the brain, activating a distributed cortical network, specifically the ventral processing pathway, during spatial cognition tasks [90], and promoting neuronal differentiation during intrauterine development, with profound effects on brain functions during postnatal life [124]. Plasma levels of IGF-1 are involved in regulating mood, depression, and cognition, stimulating neuroplasticity mechanisms [91] and exerting a protective effect against the development of Alzheimer’s disease [125].

Furthermore, the effect of androgens on brain activity must again be considered [126]. Androgen levels activate multiple hormonal regulations involving IGF-1 and 17β-estradiol. The reduction in anabolic hormones has a greater impact than a single hormonal alteration on adverse mobility outcomes in the elderly population [127,128], and this parallel decline predicts 6-year mortality in older men [129]. Testosterone therapy increases the availability of 1,25-dihydroxyvitamin D and the level of IGF-I, an essential component in the action of testosterone [128]. Furthermore, testosterone and its brain metabolites (e.g., estradiol) regulate brain physiology, cognitive abilities, and behavior [130,131]. The progressive decline in testosterone and its metabolites with aging contributes to selective memory loss and cognitive impairment [130]. A prospective longitudinal study of 4069 older men showed that low baseline testosterone levels were associated with an increased risk of dementia [132]. Testosterone administration improves cerebral perfusion in the midbrain and superior frontal gyrus [133].

Given these findings, it is crucial to assess plasma androgen and estradiol levels when analyzing the effects of GH and GF-I on brain function. The lack of plasma testosterone levels may compromise the results of clinical studies on the impact of growth hormone (GH) therapy in patients with dementia or Alzheimer’s disease. In fact, the effect of GH therapy can be completely different in patients with low plasma testosterone levels compared to those with normal levels. Both hormones are primarily responsible for integrating communication between various physiological systems that modulate cell growth and development.

## 5. Conclusions

Most studies have shown that growth hormone (GH) therapy improves cognition and memory in both healthy individuals and those with mild cognitive impairment (MCI). Only a few studies found no beneficial effects. Supraphysiological doses are more effective for clinical outcomes. GH treatment may enhance neuroregeneration and lead to improved recovery [117]. The recommended dosage is 1.5 to 4 mg/day (0.05 mg/kg/day). The treatment duration is approximately one year. Lower doses and shorter durations may produce uncertain results. Recovery of physical and mental function is particularly relevant in patients following traumatic brain injury (TBI). All studies reported significant improvements in cognitive impairment and physical condition.

A more physiological effect of GH treatment can be achieved by administering GHRH analogs, which effectively stimulate GH secretion and can attenuate age-related deficits in spatial memory [106], cognitive function [79,134], and short-term memory [79,135,136]. Some conflicting results were observed across studies. Plasma androgen levels were not assessed in the patients, which is a significant limitation, as androgens play a crucial role in maintaining brain function. However, GH therapy should be considered in patients with cognitive impairment, Alzheimer’s disease, sequelae of traumatic brain injury, and stroke.

Limitations of this study include the small number of available studies, a heterogeneous patient population, inconsistent study designs, and small sample sizes, with some studies including fewer than 10 patients. Furthermore, patients’ physical, nutritional, and clinical conditions must be considered to better understand neurological recovery. Finally, low levels of sex steroids can compromise outcomes. Nutrition, physical activity, metabolic parameters, and sex steroid levels should be included among the corrective factors. Nevertheless, the potential of GH therapy for brain regeneration is significant. A more appropriate clinical trial, conducted in a large population, that considers the influence of multiple corrective factors and includes rigorous statistical analysis is needed to validate the effect of GH on the brain.

### Safety

Regarding safety, although GH and IGFs possess mitogenic and proliferative properties, which represent a potential risk in tumor promotion and progression [137], a long-term mortality study revealed that no patients died from cancer or cardiovascular disease [138]. In a meta-analysis, Deodati et al. [139] demonstrated that there was no association between GH therapy, primarily in patients treated during childhood and adolescence, and overall mortality, cancer, or the risk of a second malignancy. When IGF-1 levels were adjusted within the normal age range, no increase in the incidence of malignancy was observed [140]. In a cohort study of patients with GH deficiency, most deaths (76%) were due to accidents or suicide, but none of the patients died from cancer or cardiovascular disease [141]. Among a total of 14,108 survivors enrolled in the Childhood Cancer Survivors Study, a retrospective cohort of childhood cancer survivors at 5 years, 361 subjects treated with GH were identified. A second malignancy was observed, but the increased risk associated with GH use appears to decrease with increasing follow-up duration [142]. In one study, GH has been used for more than 35 years, and its safety and efficacy have been extensively studied.

The effect of GH replacement therapy on the risk of secondary malignancy is smaller compared with host-related factors and tumor treatment. There is no evidence of an association between GH replacement therapy and increased cancer mortality among GH-deficient childhood cancer survivors [143].

In a meta-analysis, Shen et al. [144] evaluated the potential relationship between GH replacement therapy and the recurrence/progression of intracranial tumors. Fifteen eligible studies were included, with over 2232 cases and 3606 controls. The results showed that the recurrence/progression of intracranial tumors was not associated with GH therapy, and ependymoma was also not associated with tumor recurrence/progression. GH replacement therapy appears to be safe with regard to the risk of tumor progression.

A recent meta-analysis suggests that GH replacement therapy may reduce the risk of cancer in adults with growth hormone deficiency [145]. Bolier et al. [146] showed predominantly reassuring results regarding the safety of GH therapy in survivors, although the available information on adult cancer survivors was limited. In patients with a history of malignancy, GH therapy has not been shown to increase the risk of tumor recurrence [147].

In conclusion, the data may raise concerns about the long-term safety of GH treatment, but for short-term therapy, only minimal side effects were described, such as fluid retention [148] and carpal tunnel syndrome [149] or joint pain. No other serious side effects have been reported.

## Figures and Tables

**Table 1:** Effect of GH on memory, dementia and Alzheimer’s disease. Multiple pituitary hormone deficiency [MPHD; isolated growth hormone deficiency [IGHD; quality of life (QoL).

Authors	N. Patients Diagnosis	Age (Years)	Study Types	Duration	Dose	Effects
van Bunderen et al. 2018 [83]	32	46.6	randomized, open-label, clinical trial	1 year	dose of GH treatment based on IGF-1 target level	A high dose may impair prefrontal cognitive functioning, while a low dose may decrease vigor.
Devesa 2018 [80]	61 w No GHD MCI	1	Case study	3 weeks	0.4 mg/day	Positive effects of GH on cerebral metabolism and function related to knowledge and memory,
Baker, 2012, [79]	13,776 healthy, and 61 with MC	68	Randomized, double-blind, placebo-controlled trial.	20 weeks	GHRH 1.0 mg/d of tesamorelin	Favorable effects on cognition in both adults with MCI and healthy older adults.
Sevigny, 2008 [81]	416 AD	75.9 W 56%	double-blind, multicenter study	1 year	MK-677 25 mg	Despite 60.1% increase in serum IGF-1, levels no effect at slowing the rate of progression of AD was observed.
Sathiavageeswaran, 2007 [78]	16 GHD 18 controls	66 (60–77)	double-blind, randomized, placebo-controlled study	52 weeks	0.16+/−0.06 mg/day;	Improvement in certain measures of cognitive function in elderly patients with GHD.
Arwert et al. 2005 [84]	23 men with GHD	28.6	long-term follow-up study	10 years	0.97 mg/day	Improvement in mood and memory improved during GH therapy. Memory improved after 1 year of substitution and IGF1 levels were associated with better mood.
Oertel et al. 2004 [76]	18	21–63	double-blind, randomized placebo-controlled trial	6 months	Initial dose 6 µg/kg/day (0.125 U/kg/week). Treatment dose the double 12 µg/kg/day (0.25 U/kg/week)	Significant improvement in attentional performance. No effect on memory and non-verbal intelligence.
Stouthart et al. 2003 [74]	10 males and 10 females IGHD and MPHD	17 and 27, 5	Randomized clinical trial	12 months		Improvement in psychometric parameters and QoL.
Soares et al. 1999 [77]	9 GHD Sella tumors	39.4 (28–52)	double-blind crossover study	6 months + 6 months	0.250 IU/kg/week	Improvement in psychiatric and neuropsychological impairments of
Deijen et al. 1998 [75]	48 GHD adult men	27	double-blind cross-over study	6 months	1, 2, or 3 IU/m^2^,	Improvement in memory function in adults with CO-GHD. It does not affect psychological well-being or perceptual-motor skills.
Baum et al. 1998 [82]	40 GDH men	24–64	Randomized double-blind cross-over study	18 months	0.012+/−0.06 IU/kg per day	No significant changes in cognitive function or quality of life.
Sartorio et al. 1995 [71]	8		Case–control study	6 months		Overall improvement concerning intellectual tasks.
Bengtsson BA, 1993 [73]	10 GHD	46.5	double-blind cross-over study	26 weeks	0.25–0.5 IU/kg/week (0.013–0.026 mg/kg per day	Marked alterations in body composition, fat distribution, and bone and mineral metabolism and reduces psychiatric symptoms.
Degerblad et al. 1990 [85]	6		double-blind cross-over study	12 weeks	0.5–0.6 IU/kg/week (0.026 mg/kg per day)	No changes in isokinetic muscle strength, working capacity, mood, and cognitive function.
Almqvist et al. 1986 [72]	5	22–36	observational	8 weeks	8 IU (2.5 mg) i.m. three times weekly	Beneficial effect of GH on cognitive functions.

## Data Availability

No new data were created or analyzed in this study. Data sharing is not applicable to this article.

## Abbreviations

CNS	central nervous system
COGHD	childhood-onset growth hormone deficiency
CSF	cerebrospinal fluid
DA	Dopamine
GH	growth hormone
GHD	growth hormone deficiency
GHR	growth hormone receptors
GHSr	growth hormone secretagogues
GnRH	Gonadotropin-Releasing Hormone
HVA	homovanillic acid
IGF-1	insulin-like growth factor-1
MMSE	Mini-Mental State Examination
NMDA	N-methyl-D-aspartate
NSC	neural stem cells
Prl	prolactin
TBI	traumatic brain injury
SVZ	subventricular zone
